# Screening and brief intervention for marijuana use in Colorado

**DOI:** 10.1186/1940-0640-8-S1-A76

**Published:** 2013-09-04

**Authors:** Carolyn Swenson, Leigh Fischer, Brie Reimann

**Affiliations:** 1 HealthTeamWorks, Lakewood, CO, USA

## 

In 2000, voters in Colorado approved an amendment to the state constitution to legalize medical marijuana. In 2009, the U.S. Department of Justice indicated that patients complying with medical marijuana laws would not be a priority for prosecution. The state subsequently experienced widespread growth in in the number of individuals with a medical marijuana card and in marijuana dispensaries. In 2012 Colorado passed an amendment to legalize use of recreational marijuana. Medical and recreational marijuana legalization poses challenges for providing effective SBI. This presentation will summarize trends in marijuana use in Colorado and modifications to brief screening to improve detection of marijuana use. It will also describe challenges reported by health professionals delivering brief interventions on marijuana, and key points to emphasize in SBI training to reduce harm, prevent diversion to youth, and tailor brief interventions to individual patient conditions. Quantitative data collected through SBIRT implementation in Colorado will be summarized and qualitative data on training needs of health professionals who deliver marijuana SBI. Colorado data through 2011 indicates that approximately 8% of adults screened at-risk for problems from marijuana use, and that risky use is increasing. In a subset of patients who received SBI, six month follow-up indicated that use decreased 45%. Data collected after implementation of recreational marijuana legislation will also be presented. Health professionals need information about the following issues to provide effective SBI: how marijuana risk compares with tobacco, medication interactions and effects on chronic health conditions, driving under the influence, cyclic vomiting associated with chronic use, differential effects in youth, and a perceived lack of harm among adults and youth. Marijuana legalization poses new challenges and it is important to prepare health professionals to provide effective SBI to decrease harm and prevent diversion to youth.

